# Patients with Peyronie’s disease achieve complete plaque regression after multimodal treatment with antioxidants: a case series

**DOI:** 10.1186/s13256-022-03614-1

**Published:** 2022-10-08

**Authors:** Gianni Paulis, Giovanni De Giorgio

**Affiliations:** 1 Peyronie’s Care Center, Department of Uro-Andrology, Castelfidardo Medical Team, Castelfidardo Clinical Analysis Center, Rome, Italy; 2 Ultrasound Diagnostics Section, Department of Uro-Andrology, Castelfidardo Medical Team, Castelfidardo Clinical Analysis Center, Rome, Italy

**Keywords:** Antioxidants, Oxidative stress, Peyronie’s disease, Pentoxifylline

## Abstract

**Background:**

Peyronie’s disease is a chronic inflammatory condition of the corpora cavernosa characterized by the formation of plaque in the tunica albuginea, which results in penile deformity. Conservative medical approaches encompass oral, topical, and physical treatment. Only two cases of patients with Peyronie’s disease with complete plaque regression after treatment have been described in literature.

**Case presentation:**

*Case 1*: A 50-year-old Caucasian man with penile pain and double penile curvature of 5° (left ventrolateral), palpable nodule, and normal penile rigidity. The patient underwent multimodal therapy (oral antioxidants + topical diclofenac gel). At follow-up after over 4 years of treatment, the patient no longer complained of any penile deformity or pain. Ultrasound examination did not show any plaque. *Case 2*: A 26-year-old Caucasian man with lateral-right penile curvature of 30° (previous congenital curvature of 15°), palpable nodule, and normal penile rigidity. The patient underwent multimodal therapy (oral antioxidants + topical diclofenac gel + penile injections/pentoxifylline). After 28 months of treatment, the patient presented a lateral right curve of 15° at follow-up, similar to the original congenital penile curvature. Ultrasound examination no longer showed any plaque. *Case 3*: A 36-year-old Caucasian man with penile pain and a complex penile curvature of 15° and 20° (left dorsolateral), palpable nodule, and normal penile rigidity. The patient underwent multimodal therapy (oral antioxidants + topical diclofenac gel + penile injections/pentoxifylline). At follow-up after 28 months of treatment, the patient presented a dorsal curve (10°) similar to the original congenital curvature. Penile palpation did not detect any nodules, and ultrasound no longer showed any plaque.

**Conclusions:**

This study demonstrates that our multimodal therapy is able to completely regress plaque, as demonstrated in our previously published article. Peyronie’s disease has the potential to be treated conservatively with good results. However, this method of treatment needs to be combined with accurate ultrasound assessment, performed using a sufficiently advanced machine by an experienced operator.

## Background

Peyronie’s disease (PD) is a chronic inflammatory condition of the corpora cavernosa characterized by the formation of fibrous or calcified plaque in the tunica albuginea, resulting in penile deformity (for example curvature, divots, hourglass deformity). Patients suffering from PD present with penile pain (20–70%), penile deformity (94%), and erectile dysfunction (over 30%) [1, 2]; depression (48%) [3]. Although the origin of PD is still not completely understood, trauma—including micro-injuries—is postulated to be its most likely cause [4]. As fibrin accumulates in the site of the trauma, it triggers an inflammatory response involving an overproduction of fibrogenic cytokines and free radicals [5–8]. Conservative approaches encompass oral therapy with colchicine, potassium *para*-aminobenzoate, tamoxifen, antioxidants, phosphodiesterase-5 inhibitors, and so on; topical therapy consisting of injections with verapamil, corticosteroids, *Clostridium histolyticum* collagenase, interferon-α2b (IFNa2b), hyaluronic acid, pentoxifylline (PTX); and physical therapy (vacuum devices and/or penile traction devices, iontophoresis, extracorporeal shock wave therapy (ESWT), and so on) [9, 10]. A multimodal treatment combines various therapeutic agents, as well as different forms of administration (for example oral therapy and injections), and does not rule out concurrent use of physical treatment options such as iontophoresis, penile extenders, vacuum devices, and ESWT. A combination of treatment approaches can offer better outcomes than a single substance or therapy [11].

This paper presents three cases of PD patients treated with a multimodal antioxidant therapy (including oral antioxidants, topical diclofenac gel + penile-injections/pentoxifylline) at our Peyronie’s Care Center. The three patients achieved complete regression of the area affected by disease. This is the second article describing the complete plaque regression achieved in patients with PD.

## Case reports

### Case 1

A 50-year-old Caucasian male, non-smoker, with normal body weight, presented to our clinic in July 2014 complaining of mild penile pain during erection and penile curvature with onset about 6 months before. The visual analogue pain score (VAS) was 2 (score from 0 to 10). Subjective assessment of erection, evaluated using the International Index of Erectile Function (IIEF) questionnaire, provided a score of 27. When administering the IIEF questionnaire, we took into consideration questions 1, 2, 3, 4, 5, and 15, which specifically refer to *erectile function* (normal range from 26 to 30). The penile deformity consisted of both a ventral curvature, with a 5° angle, and a lateral left curvature of 5°. On palpation at the distal third of the penis, physical examination revealed a nodule measuring about 20 mm in length. The patient underwent a physical examination and penile Doppler ultrasound (alprostadil 10 mcg). The volume of the plaque was measured in its three dimensions using the ellipsoid formula (volume = 0.524 × length × width × thickness) [12, 13].

**Fig. 1:**
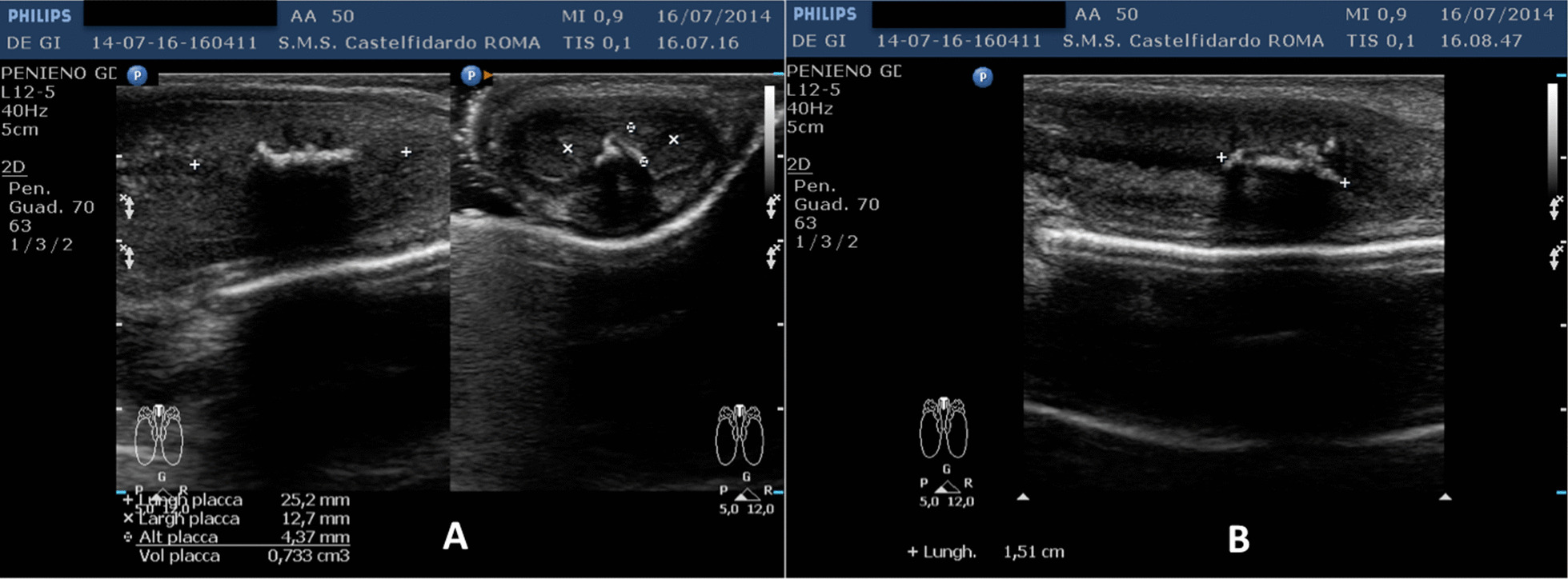
Penile ultrasound prior to treatment. **A** Plaque measurement in longitudinal and transverse scan. **B** Calcification measurement in longitudinal scan

Cavernous artery flow and end-diastolic velocity were normal: peak systolic velocity was 74 cm/seconds (bilaterally); end-diastolic velocity was 0 cm/seconds (bilaterally). The penile plaque was located ventrally and at the distal third of the penis; its ultrasound aspect was iso-hypoechoic and it measured 25.2 × 12.7 × 4.37 mm (volume = 733 mm^3^). Within the plaque there was a calcification measuring 15.1 × 4.0 mm (Fig. 1, see Table 1 ).

**Table 1.** Case summaries of three patients with Peyronie’s disease treated by combined multimodal therapy

No.	Patient age	Comorbidities	Penile plaque site	Ultrasound measurements (length × width × thickness) and plaque volume (A) before and (B) after treatment	Type of deformity (A) initial and (B) after treatment	Pain score/VAS scale (1–10)	IIEF score before and after treatment	Total duration of treatment until plaque regression	Complete combined multimodal treatment
1	50 years	None	Distal third	(A) 25.2 × 12.7 × 4.37 mm with inner calcification, 15.1 × 8.0 mm volume = 733 mm^3^ (B) No plaque detected	A) 5° ventral curvature + 5° left curvature (B) None	VAS score = 2 disappeared after 12 months	27 > 29	53 months	*Orally* Propolis 600 mg + bilberry 160 mg + silymarin 400 mg + ginkgo biloba 250 mg + L-carnitine 1000 mg + coenzyme Q-10 100 mg + Boswellia 200 mg + vitamin E 30 mg/daily/for 53 months + *topically* diclofenac gel 4%/2 × daily/for 53 months* The patient refused peri-plaque penile injections with pentoxifylline
2	26 years	Congenital (15°) right penile curvature	Proximal third	(A) 10.1 × 7.65 × 3.02 mm volume = 122 mm^3^ (B) No plaque detected	(A) 30° right penile curvature (B) 15° right penile curvature (previous condition = congenital lateral right penile curvature)	VAS score = 0	28 > 29	28 months	*Orally*: Propolis 700 mg + bilberry 180 mg + silymarin 400 mg + ginkgo biloba 240 mg + L-carnitine 1000 mg + coenzyme Q-10 100 mg + Boswellia 200 mg + vitamin E 48 mg + vitamin C 50 mg + superoxide dismutase 11,000 IU/g 10 mg/daily/for 28 months + *topically* diclofenac gel 4%/2 × daily/for 28 months + *peri-plaque penile injections* pentoxifylline 100 mg (30 G needle) every month for 12 months, and then one penile injection every 2 months for 12 months (18 total injections)
3	36 years	Congenital (10°) dorsal penile curvature, lichen sclerosus, chronic prostatitis	Middle third	(A) 20.7 × 13.3 × 3.78 mm volume = 543 mm^3^ (B) No plaque detected	(A) 15° dorsal penile curvature + 20° left penile curvature (B) 10°e dorsal penile curvature (previous condition = congenital dorsal right penile curvature)	VAS score = 5 disappeared after 12 months	25 > 29	44 months	*Orally*: Propolis 700 mg + bilberry 180 mg + silymarin 400 mg + ginkgo biloba 240 mg + L-carnitine 1000 mg + coenzyme Q-10 100 mg + Boswellia 200 mg + vitamin E 48 mg + vitamin C 50 mg + superoxide dismutase 11,000 IU/g 10 mg/daily/for 44 months + *topically* diclofenac gel 4%/2 × daily/for 44 months + *peri-plaque penile injections* pentoxifylline 100 mg (30 G needle) every 2 weeks for 6 months, and then one penile injection every month for 12 months, and then one penile injection every 2 months for 12 months (30 total injections)

*IIEF* International Index of Erectile Function, *VAS* visual analogue pain score

To obtain informed consent, the patient was notified of the necessary length of treatment owing to the presence of a chronic disease. The patient did not consent to the publication of his penis photos, even if anonymized. Beginning in July 2014, after giving his informed consent, the patient underwent the following treatment: Combined therapy with oral antioxidants propolis 600 mg + bilberry 160 mg + silymarin 400 mg + ginkgo biloba 250 mg + L-carnitine 1000 mg +  coenzyme Q10 100 mg + Boswellia 200 mg + vitamin E 30 mg/daily/ + opical diclofenac gel 4%/2 × daily for 12 months.

Oral antioxidants were contained in the following products: Propolifix Plus capsules (propolis + ginkgo biloba), Silifix Plus capsules (silymarin + boswellia + bilberry), Carnitin E Q10 sachets (L-carnitine + coenzyme Q10 + vitamin E).

The patient refused to be treated with penile infiltrations for fear of pain.

Follow-ups were scheduled approximately every 12 months, always confirming the same therapy for each treatment cycle.

After four treatment cycles (each lasting 12 months), the plaque was 99.3% smaller than its initial volume, and the calcification within the plaque was further reduced in size.

Considering the good results, we decided to continue the same treatment for the next 5 months.

After 5 months of antioxidant treatment, and after approximately 4 years and 5 months of multimodal treatment, at follow-up, the IIEF score was 29, penile palpation did not detect any nodule, the two original curvatures were observed to have disappeared, and ultrasound examination no longer showed any plaque (Fig. 6). The multimodal treatment with antioxidants was therefore suspended. The patient did not report any side effects after the treatment.

The patient shared with us satisfaction for the excellent result of the treatment received.

The same ultrasound machine was used at every examination (Philips HD 15) until the follow-up after the third therapy cycle when the ultrasound system in our clinic had been replaced by a new machine (Philips Affinity 70 G). The same doctor performed all ultrasound examinations at every follow-up.

**Fig. 2:**
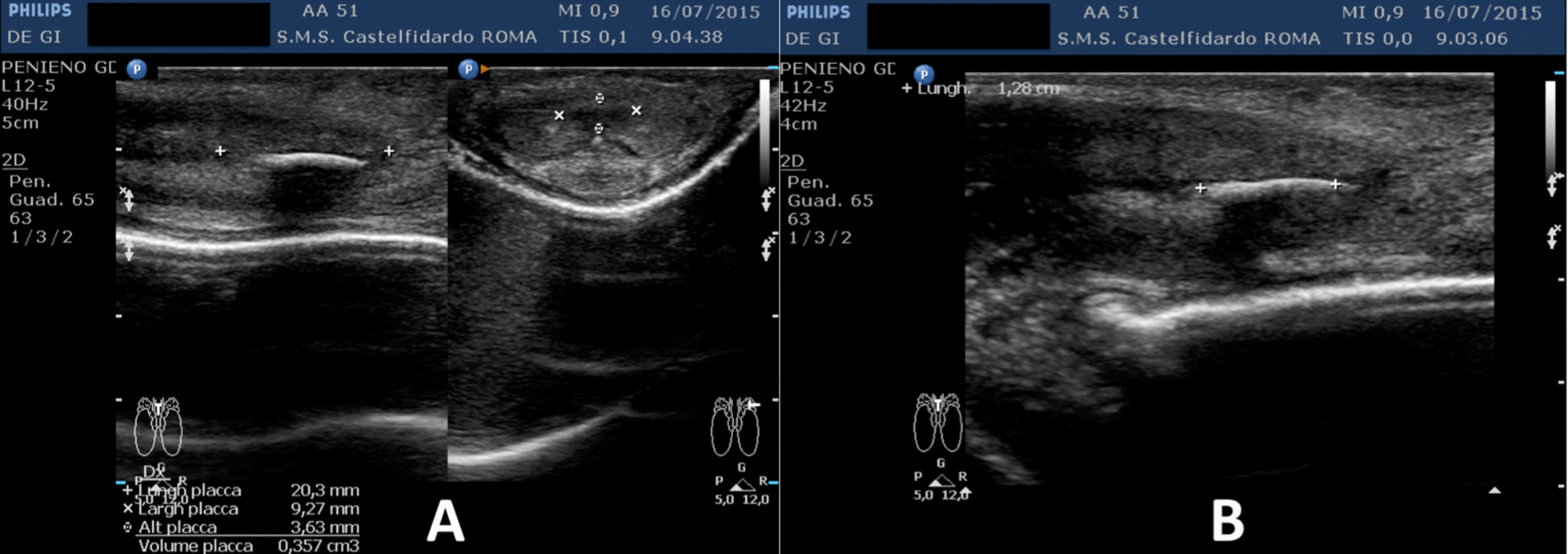
Penile ultrasound after the first treatment cycle (12 months). **A** Plaque measurement in longitudinal and transverse scan. **B** Calcification measurement in longitudinal scan

**Fig. 3:**
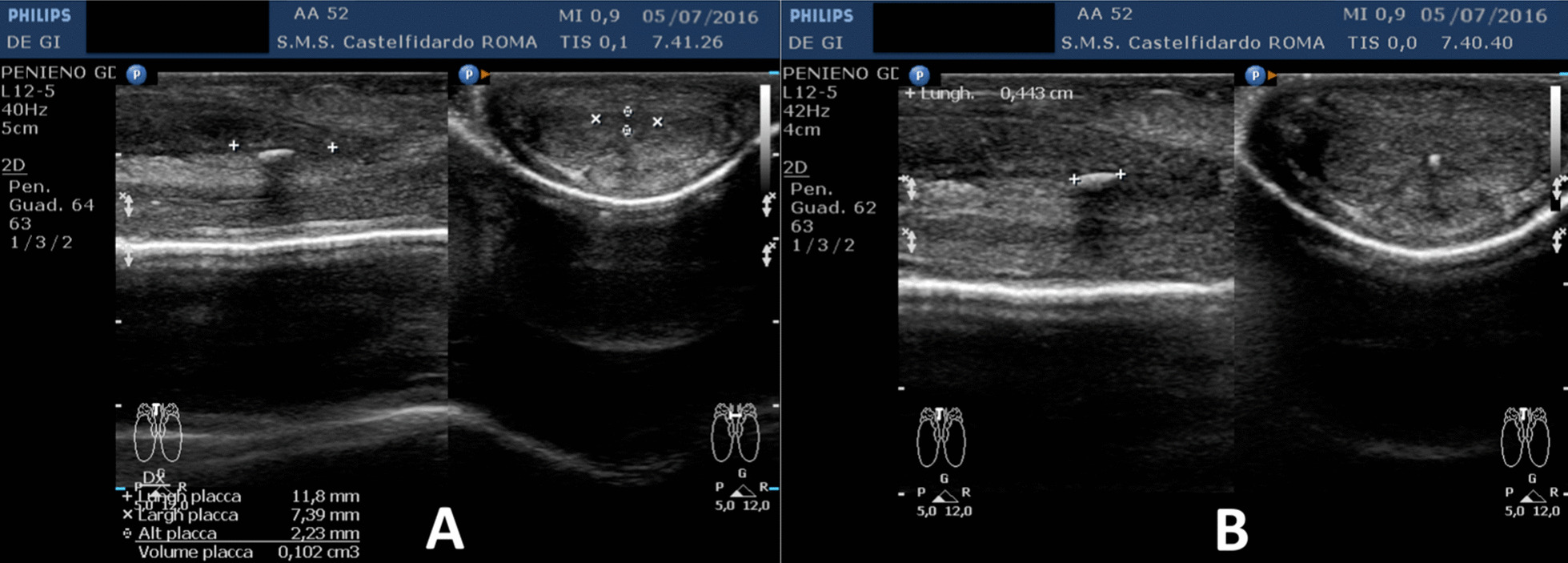
Penile ultrasound after the second treatment cycle (12 months). **A** Plaque measurement in longitudinal and transverse scan. **B** Calcification measurement in longitudinal scan

**Fig. 4:**
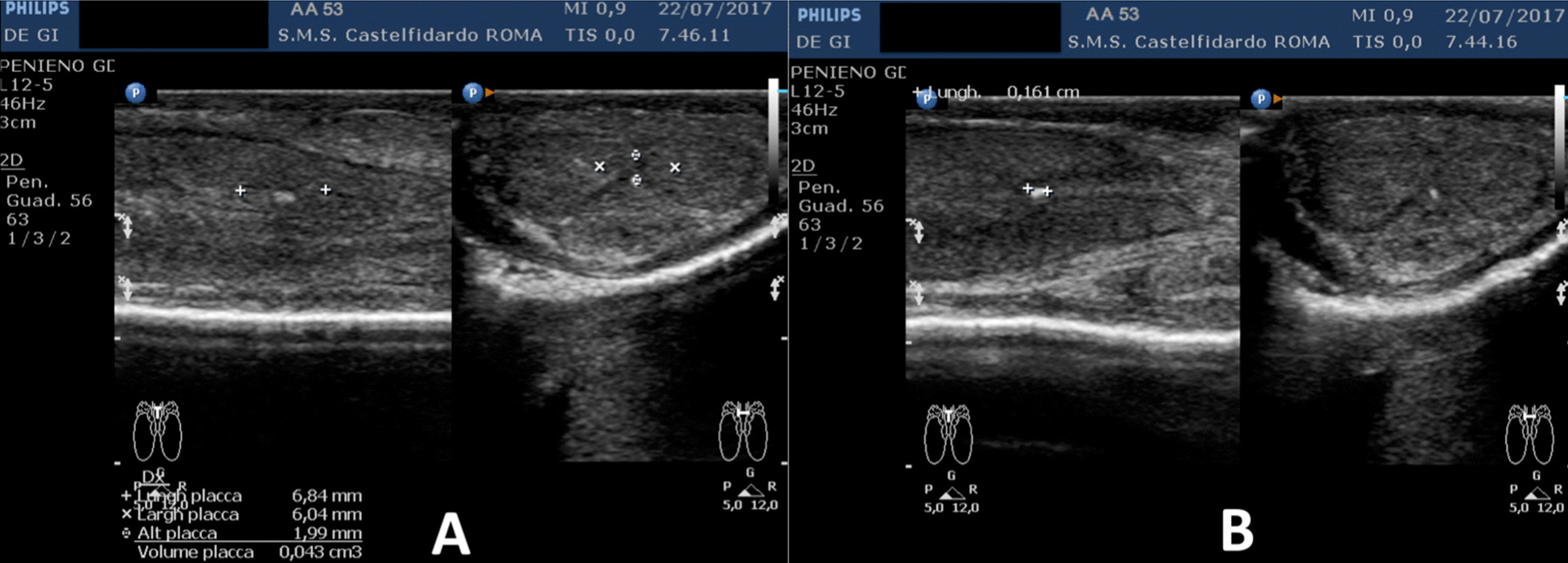
Penile ultrasound after the third treatment cycle (12 months). **A** Plaque measurement in longitudinal and transverse scan. **B** Calcification measurement in longitudinal scan

**Fig. 5:**
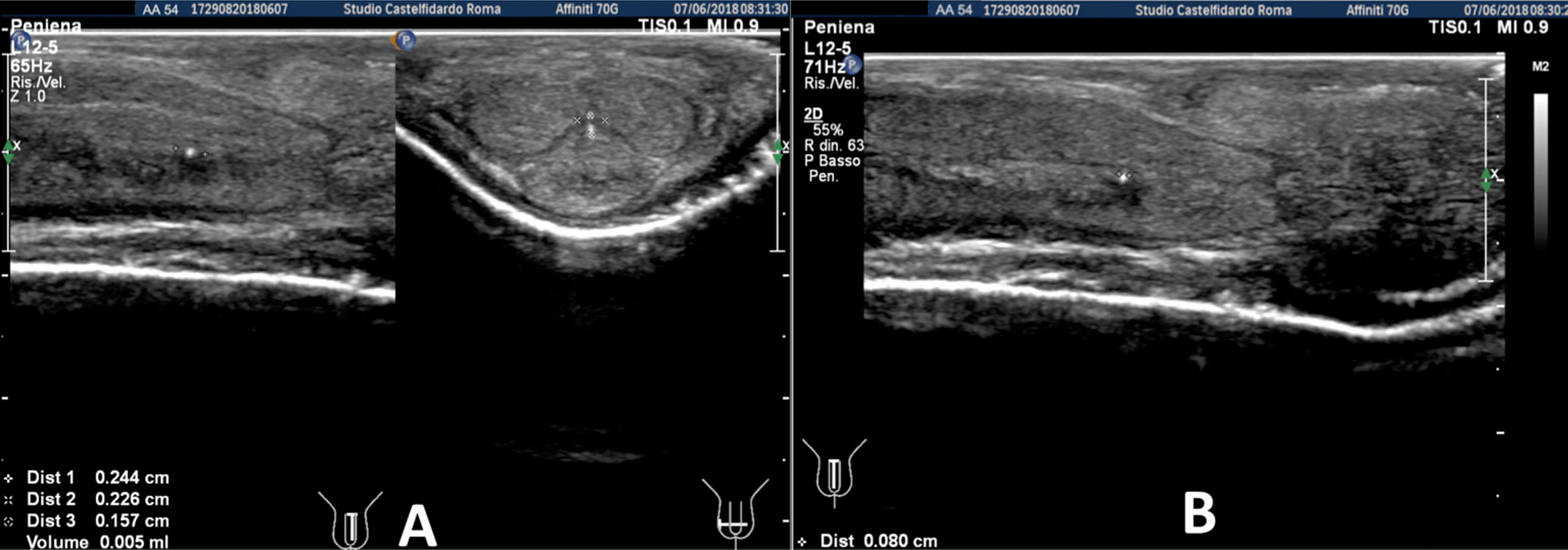
Penile ultrasound after the fourth treatment cycle (12 months). **A** Plaque measurement in longitudinal and transverse scan. **B** Calcification measurement in longitudinal scan

**Fig. 6:**
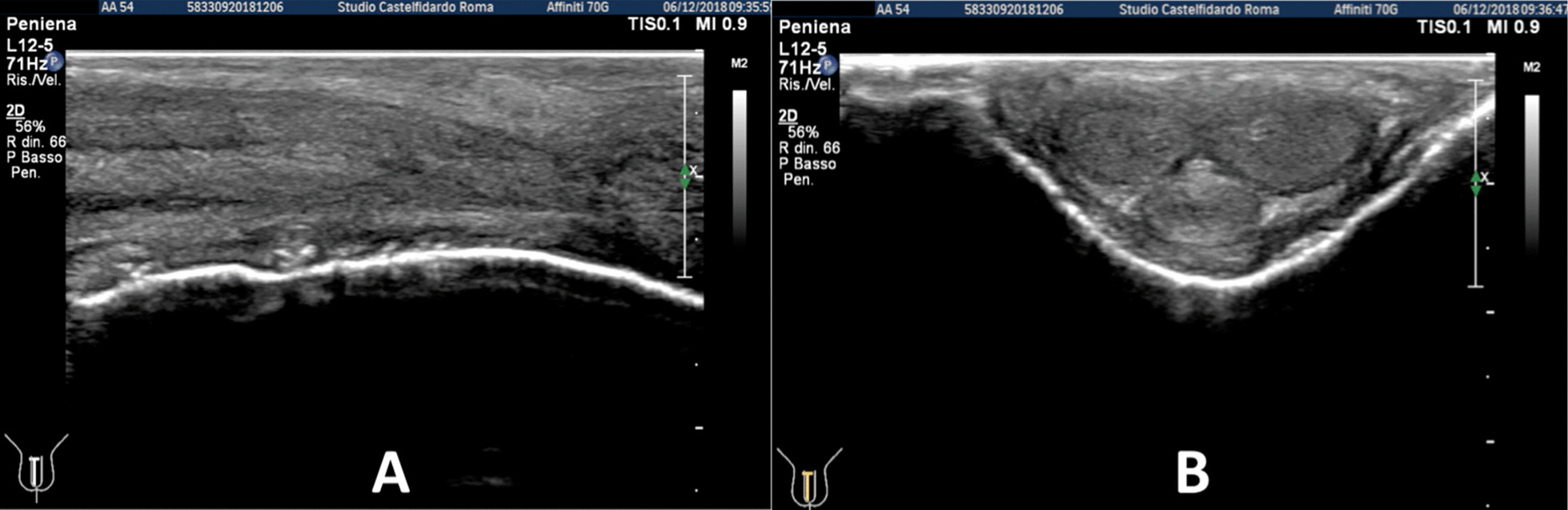
Penile ultrasound after the fifth treatment cycle (5 months). **A** Longitudinal scan. **B** Transverse scan

Progressive improvements (plaque volume reduction) after each cycle are shown in Figs. 2, 3, 4, 5, and 6. (See also Table 1).

### Case 2

**Fig. 7:**
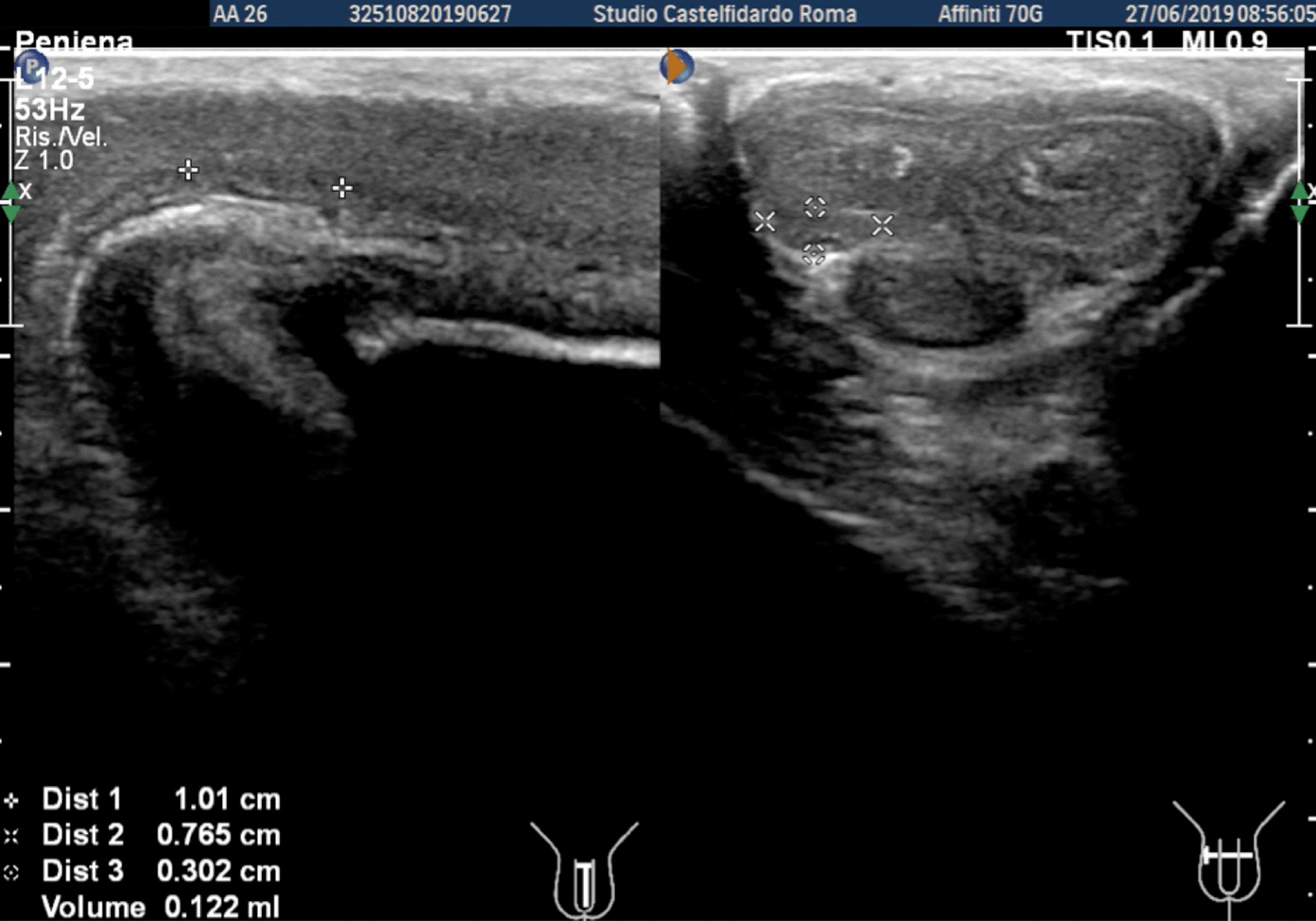
Penile ultrasound and plaque measurement prior to treatment (longitudinal and transverse scan).

A 26-year-old Caucasian man with congenital lateral-right penile curvature (15°) before the onset of PD, presented to our clinic in June 2019; he did not report any penile pain, but complained a worsening of penile curvature with onset about 6 months previously. At the time of our observation, the patient presented a lateral-right penile curvature of 30°. The patient was therefore asked to fill in the IIEF-questionnaire on erectile function, and underwent physical examination and penile Doppler ultrasound (alprostadil 10 mcg). The IIEF score was 28 (normal range 26–30). On penile palpation during physical examination, no nodules were detected. Cavernous artery flow and end-diastolic velocity were normal: peak systolic velocity = 98 cm/seconds on the right and 96 cm/seconds on the left; end-diastolic velocity = 0 cm/seconds bilaterally. The penile plaque, located at the proximal third of the penis, had an iso-hypoechoic appearance, and its dimensions were 10.1 × 7.65 × 3.02 mm, with a volume of 122 mm^3^ (Fig. 7, see Table 1 at the end of the Case report section).

To obtain informed consent, the patient was notified of the necessary length of treatment owing to the presence of a chronic disease. The patient did not consent to the publication of his penis photos, even if anonymized. After receiving the patient’s informed consent, we began the following treatment in July 2019: Combined therapy with oral antioxidants propolis 700 mg + bilberry 180 mg + silymarin 400 mg + ginkgo biloba 240 mg + L-carnitine 1000 mg + coenzyme Q10 100 mg + Boswellia 200 mg + vitamin E 48 mg + vitamin C 50 mg + superoxide dismutase 11,000 IU/g 10 mg/daily + topical diclofenac gel 4%/2 × daily + peri-plaque penile injection of pentoxifylline 100 mg with 30 G needle every month for 12 months.

All oral antioxidants were contained in the following product: Peyflog tablets.

Follow-ups were scheduled approximately every 12 months, always confirming the same therapy for each treatment cycle. Considering the good response to the first multimodal treatment, we decided to schedule a second cycle of oral and topical treatment for 12 months, using the same agents and doses and reducing the frequency of peri-plaque penile injections with pentoxifylline 100 mg to one penile injection every 2 months for 12 months.

At the end of the second cycle of treatment and after 28 months of multimodal treatment, the patient underwent follow-up with physical examination and penile Doppler ultrasound. At follow-up, we observed a further reduction in the angle of the lateral right curve, which measured 15°, similar to the original congenital penile curvature, before the onset of PD. Ultrasound examination no longer showed any plaque (Fig. 9).

Our multimodal treatment with antioxidants was therefore suspended.

The patient shared with us satisfaction for the excellent result of the treatment received.

The patient did not report any side effects after the treatment.

The same ultrasound machine was used at initial presentation and in the follow-up examinations (Philips Affinity 70 G), and the ultrasound examination was always performed by the same doctor.

**Fig. 8:**
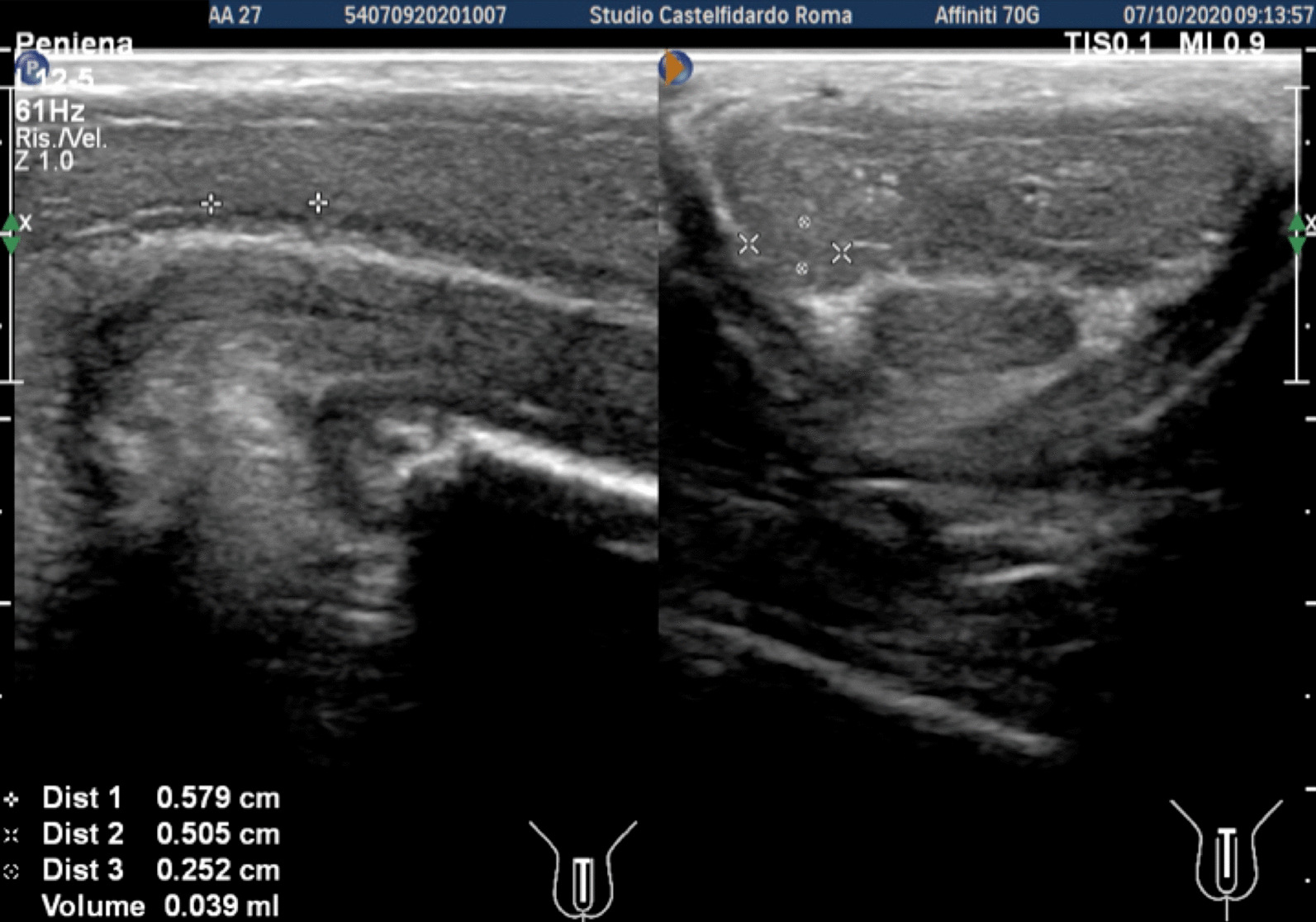
Penile ultrasound after the first treatment cycle (longitudinal and transverse scan)

**Fig. 9:**
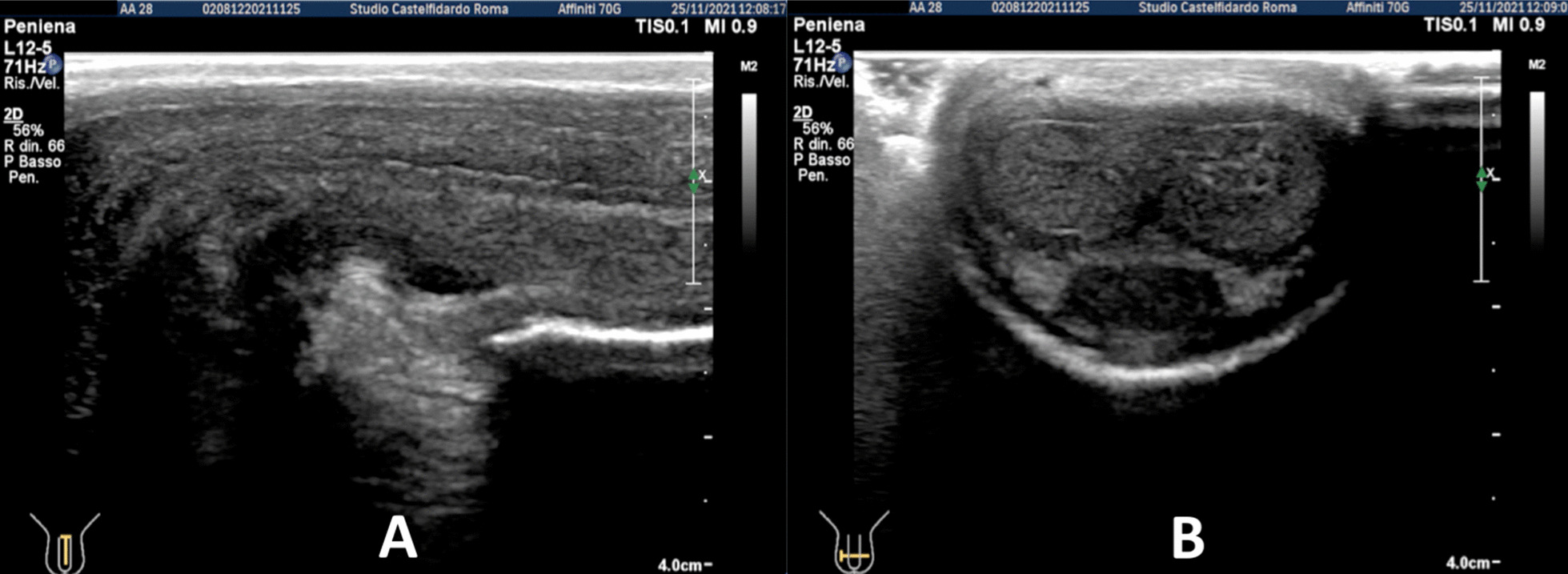
Penile ultrasound after the second treatment cycle. **A** longitudinal scan. **B** Transverse scan

Progressive improvements (plaque volume reduction) after each cycle are shown in Figs. 8 and 9. (See also Table 1)

### Case 3

A 36-year-old Caucasian man with chronic prostatitis, lichen sclerosus, and congenital dorsal penile curvature (10°) presented to our clinic in April 2018, complaining of penile pain during erection within the last 18 months, and new complex penile curvature with onset about 12 months previously. At the time of our observation, the patient presented the penile deformity of both a dorsal curvature with a 15° angle and a lateral left curvature of 20°. The VAS was 5 (score from 0 to 10). The patient was therefore asked to fill in the IIEF-questionnaire on erectile function, and underwent physical examination and penile Doppler ultrasound (alprostadil 10 mcg). The IIEF score was 25 (normal range: 26–30). On palpation, physical examination revealed a nodule measuring about 20 mm in length at the middle third of the penis.

**Fig. 10:**
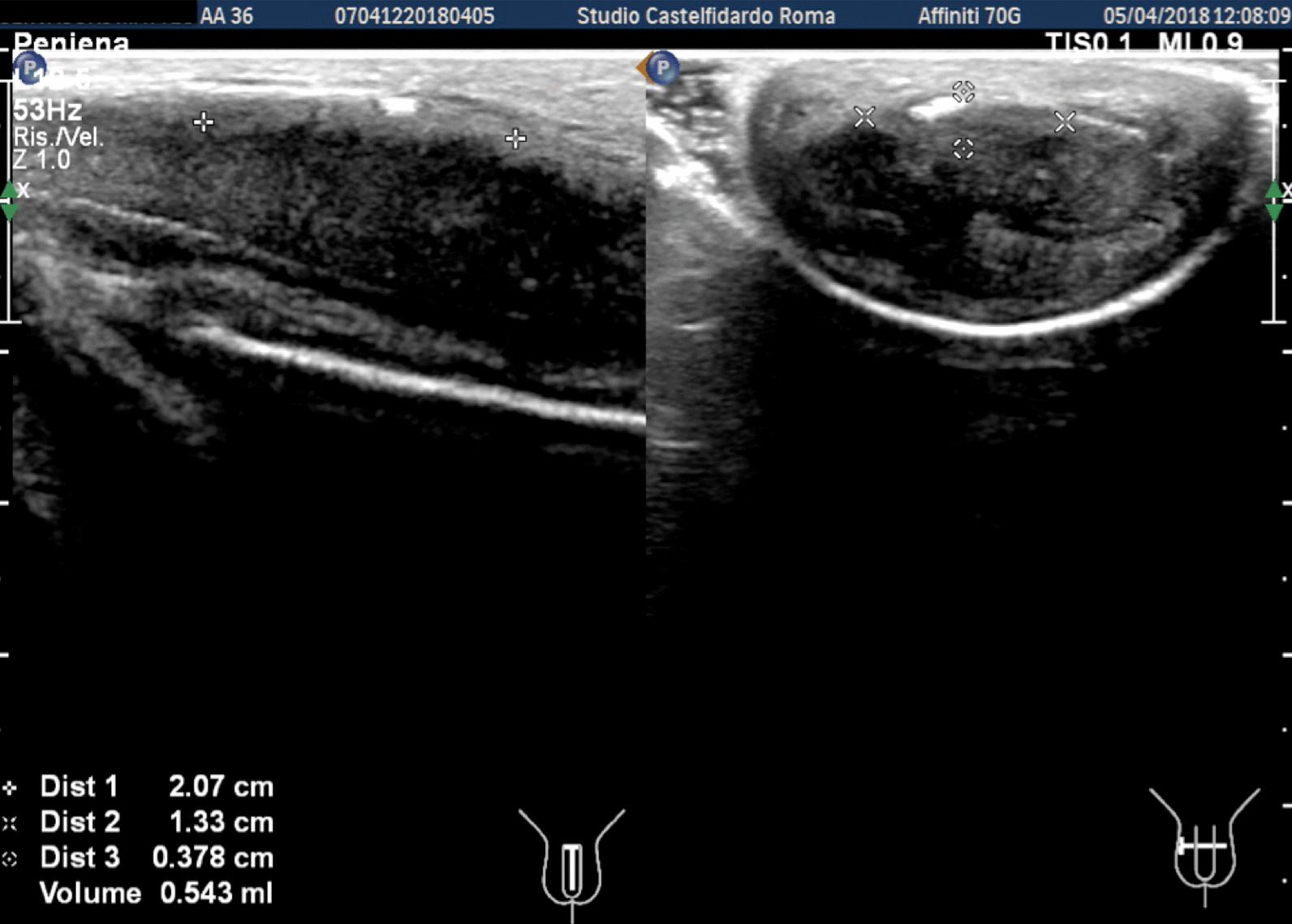
Penile ultrasound and plaque measurement prior to treatment (longitudinal and transverse scan)

Cavernous artery flow and end-diastolic velocity were normal: peak systolic velocity = 92 cm/seconds (bilaterally), end-diastolic velocity = 0 cm/seconds bilaterally. The penile plaque, located at the middle third of the penis, had a hypo-isohyperechoic appearance and its dimensions were 20.7 × 13.3 × 3.78 mm, with a volume of 543.0 mm^3^. Within the plaque there was a calcification measuring 4.2 × 3.7 mm (Fig. 10, see Table 1 at the end of the Case report section).

To obtain informed consent, the patient was notified of the necessary length of treatment owing to the presence of a chronic disease. The patient did not consent to the publication of his penis photos, even if anonymized. After receiving the patient’s informed consent, we began the following treatment in July 2019: Combined therapy with oral antioxidants propolis 700 mg + bilberry 180 mg + silymarin 400 mg + ginkgo biloba 240 mg + L-carnitine 1000 mg + coenzyme Q10 100 mg + Boswellia 200 mg + vitamin E 48 mg + vitamin C 50 mg + superoxide dismutase 11,000 IU/g 10 mg/daily + topical diclofenac gel 4%/2 × daily + peri-plaque penile injection (pentoxifylline 100 mg) every 2 weeks for 6 months. All oral antioxidants were contained in the following product: Peyflog tablets.

Considering the good response to the first multimodal treatment, we decided to schedule subsequent treatment cycles of oral and topical treatment for 24 months, using the same agents and doses and reducing the frequency of peri-plaque penile injections with pentoxifylline 100 mg to one penile injection every month for 12 months (second cycle) and subsequently, we further reduced the frequency of penile injections to one injection every 2 months for 12 months (third cycle).

Considering the good response to the the third cycle of multimodal treatment, we decided to schedule a fourth treatment cycle with the oral and topical treatment, using the same agents and doses for 6 months, and to suspend the peri-plaque pentoxifylline injections.

After 6 months of oral and topical treatment, the patient underwent the same follow-up. The IIEF score was 26. We observed a dorsal curvature with a 10° angle, similar to the original congenital penile curvature. Palpation no longer detected the presence of the nodule. Ultrasound examination no longer showed any plaque (Fig. 14, Table 1).

The patient was not accurate in meeting the follow-up deadlines, and so after approximately 3 years and 8 months of multimodal therapy with antioxidants, the treatment was suspended. The patient did not report any side effects after the treatment.

The patient shared with us satisfaction for the excellent result of the treatment received.

The same ultrasound machine was used at initial presentation and in the follow-up examinations (Philips Affinity 70 G), and the ultrasound examination was always performed by the same doctor.

**Fig. 11:**
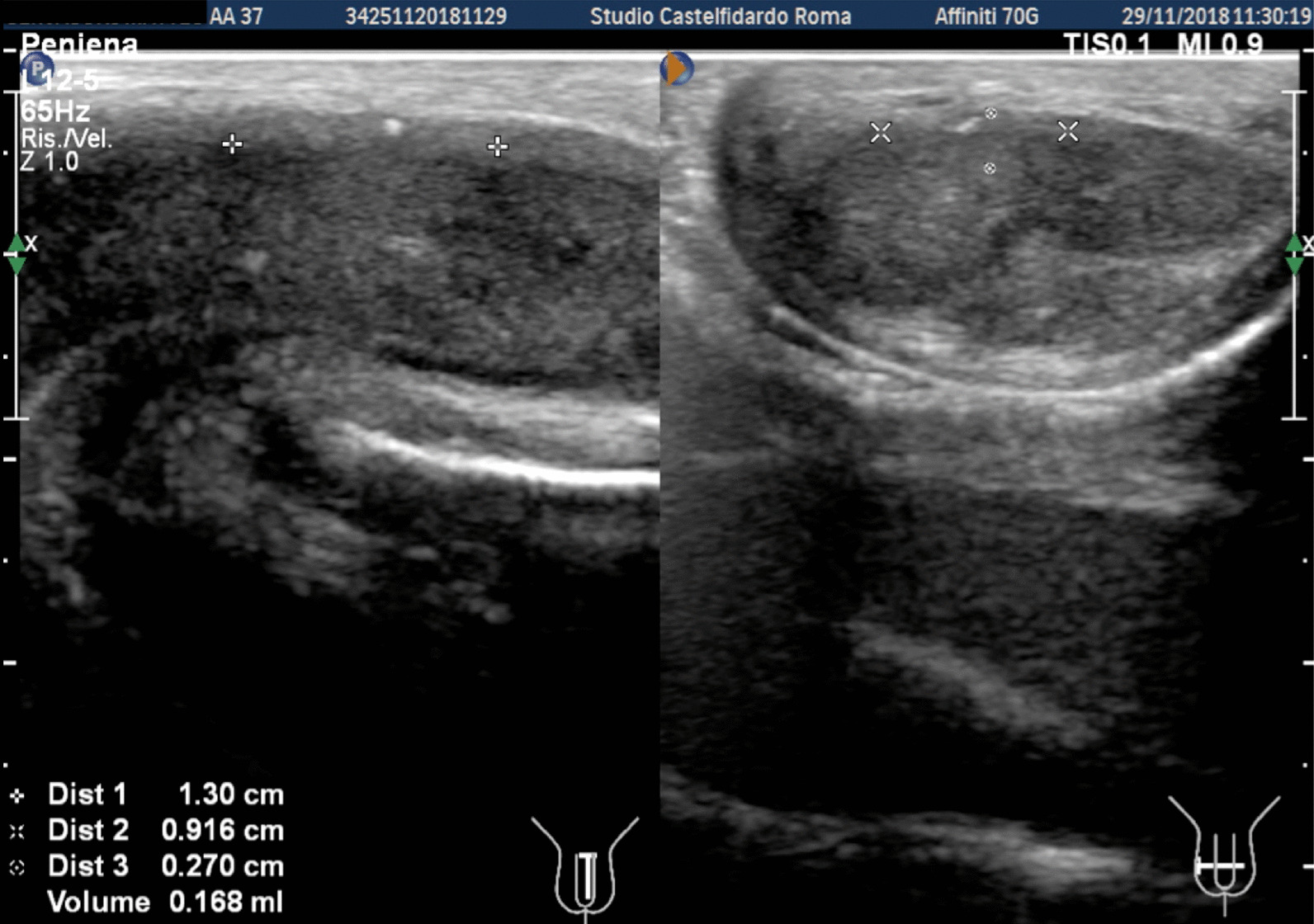
Penile ultrasound after the first treatment cycle (longitudinal and transverse scan)

**Fig. 12:**
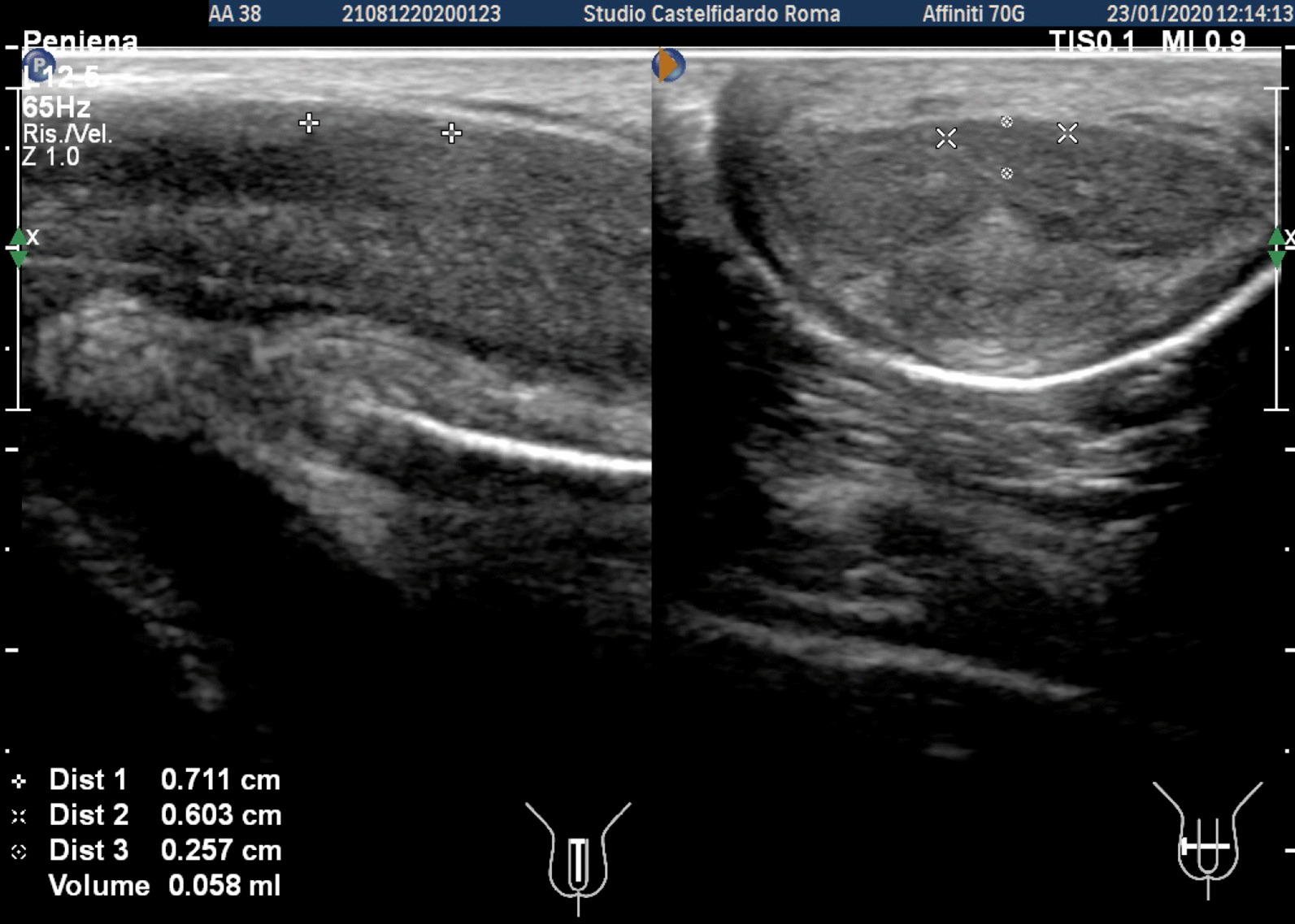
Penile ultrasound after the second treatment cycle (longitudinal and transverse scan)

**Fig. 13:**
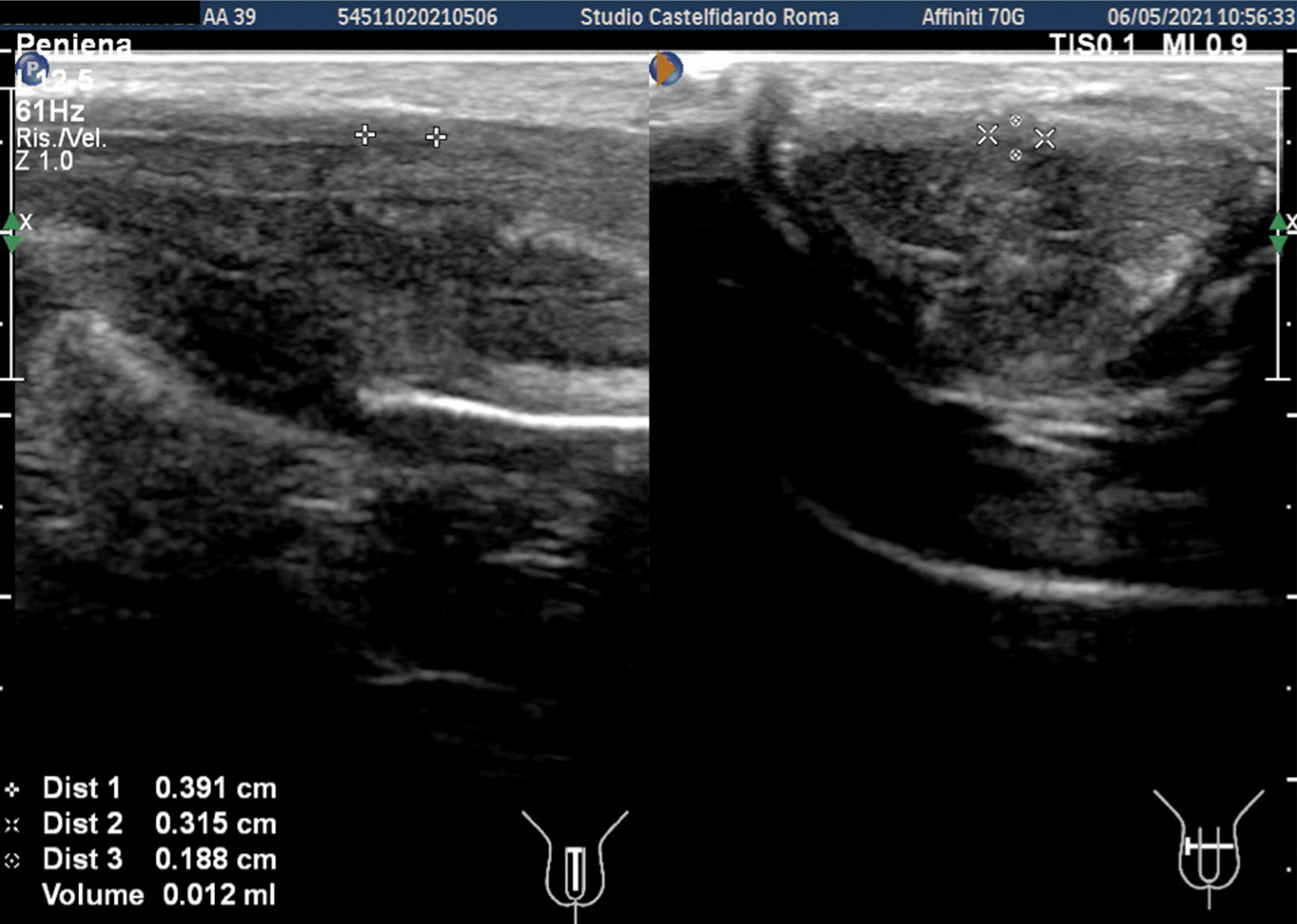
Penile ultrasound after the third treatment cycle (longitudinal and transverse scan)

**Fig. 14:**
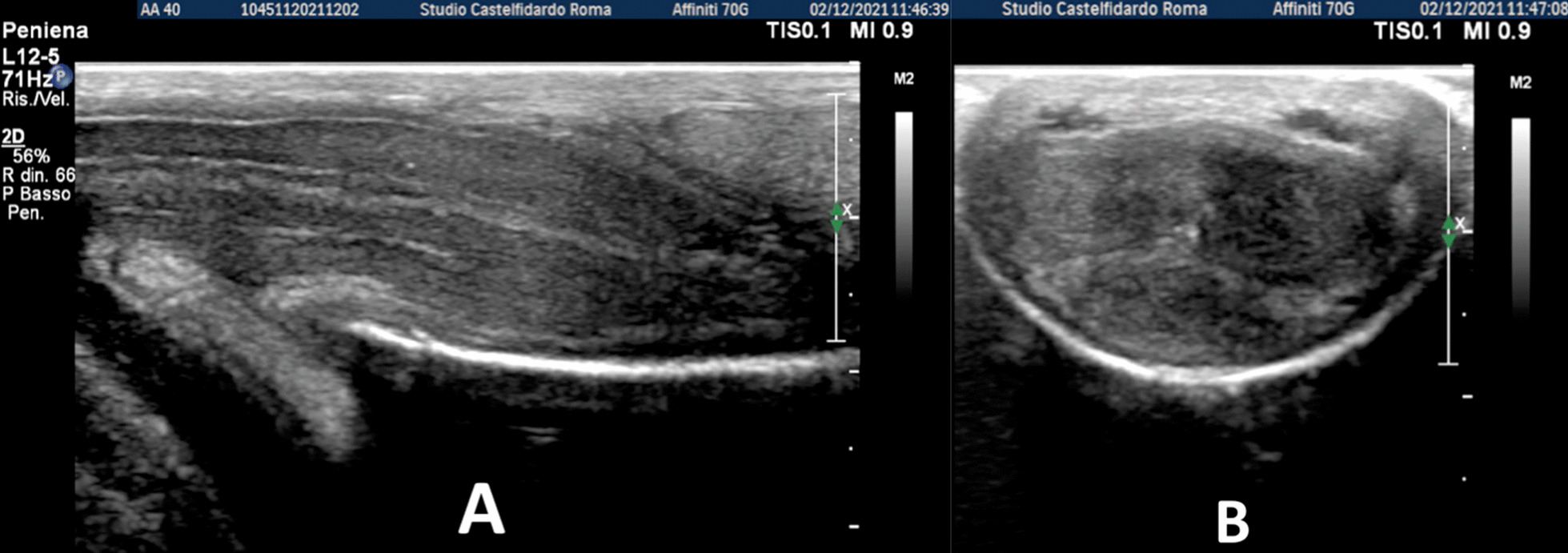
Penile ultrasound after the fourth treatment cycle. **A** longitudinal scan. **B** Transverse scan

Progressive improvements (plaque volume reduction) after each cycle are shown in Figs. 11, 12, 13, and 14. (See also Table 1)

## Discussion

It has been observed in the literature that, following multimodal treatment with antioxidants in patients with PD, there may be a reduction in plaque, as well as an improvement in the corresponding deformity [14–22].

We know that chronic inflammatory diseases (including PD) are very long lasting, therefore, an adequate period of treatment is required to stop the course of the disease and for progressive reabsorption of the plaque; considering this, the multimodal treatment of PD will necessarily be long (a few years), as described in our case reports.

Probably in *Case 1* the treatment was necessarily longer because this patient refused the PTX injections. This is the second article describing the complete plaque regression achieved in human patients with PD [23]. In the literature, there is one experimental study on rats where complete plaque regression has been achieved [24].

Compared with our previous case report, in this article the antioxidant therapy is significantly enhanced, in fact in all three cases new antioxidant substances have been added (L-carnitine, coenzyme Q-10, Boswellia) and specifically in cases 2 and 3, we increased the dosages of two substances (propolis from 600 to 700 mg and bilberry from 160 to 180 mg), and we also added other substances (vitamin C 50 mg + superoxide dismutase 11,000 IU), which allowed us to significantly reduce the dosage of vitamin E.

In addition, in this new article, there is a case report of a patient (Case 2, 26 years old) who had regression of the plaque in a significantly shorter time, most likely due to his younger age. Compared with the older PD patients, younger patients present themselves early to the medical examination, and therefore in an early stage of the disease [25].

In fact, in this case, at the ultrasound study the plaque had an iso-hypoechoic appearance and with smaller dimensions (122 mm^3^) than all the other cases treated in this article and in the previous case report.

At our Peyronie’s Care Center, we treat many PD patients with similar “combination” therapies. We decide on the most suitable treatment for each patient, taking into account the size of the plaque, the presence of any associated erectile dysfunction, the age of the patients, and their associated risk factors.

Our indications for conservative treatment with multimodal therapy are that Peyronie’s disease is in the active (not stabilized) phase and that the plaque must not be completely calcified.

The associated risk factors (erectile dysfunction, diabetes mellitus, cigarette smoking, arterial hypertension, and so on) require longer treatment to obtain a good result.

In our current experience, we are treating about 50 PD patients with similar multimodal therapy. Other patients (about 15), despite the planned follow-up, showed a reduction in plaque and penile curve, but dropped out of treatment because, in their opinion, it was too long. The negative results we have obtained are from some patients (about 10 cases, owing to important associated risk factors), in whom the cure was longer before obtaining the final result.

Despite the limited number of patients, we believe that this experience is very important and suggestive for all Uro-Andrologists. We think disease regression was made possible by the properties of the antioxidants used. These antioxidants are known to be capable of interrupting the inflammatory process by combating oxidative stress, an essential player in PD pathogenesis. All substances in our combined approach (propolis, bilberry, carnitine, coenzyme Q10, silymarin, ginkgo biloba, vitamin C, vitamin E, and diclofenac) are factor NF-kB inhibitors and block production of proinflammatory cytokines [8, 26–40]. We believed from the start that topical treatment with diclofenac would be useful in the treatment of PD, for its well-established painkilling and antiinflammatory properties, as well as its free-radical scavenging activity and action against the proinflammatory cytokine cascade, including factor NF-kB production. Diclofenac has also been proven to be capable of being absorbed topically, not just subcutaneously but in subfascial tissues. Radermacher *et al*. (1991) also proved that topical diclofenac gel is able to penetrate into the articular capsule of the knee, which is much thicker than the tunica albuginea of the penis [41].

Throughout our entire treatment, the oral and topical therapy did not vary. In cases 2 and 3, at each subsequent treatment cycle, the frequency of peri-plaque injections with PTX 100 mg was gradually reduced. This decision was based on the fact that disease progression had already stopped after the first cycle of treatment, and signs of partial regression were observed on physical examination and diagnostic imaging. Furthermore, as trauma is undoubtedly at the origin of PD and even micro-injuries may bear serious consequences in genetically predisposed patients, even injections must be considered a source of microtrauma for the tissues, therefore we made sure to use a very thin needle when performing the injections in the area around the plaque, and we decreased the number of injections at every treatment cycle.

In the literature, ultrasound imaging of PD has been described by many as being incapable of providing accurate plaque measurements [42]. On the contrary, we believe that an ultrasound examination performed with a cutting-edge ultrasound machine by a highly experienced operator with extensive knowledge of the disease can provide a measurement of the plaque that is very helpful to assess patients at presentation and to accurately evaluate treatment outcome at follow-up [43, 44].

## Conclusion

Many urologists think that Peyronie’s disease is an incurable disease, as some authors have written. The result is a renunciative therapeutic attitude of many urologists. Despite the limited number of patients, this study shows that a multimodal combined antioxidant treatment can yield good outcomes. Hence, PD can be cured.

In addition to the multimodal treatment, we believe a key element is a correct ultrasound assessment performed with a state-of-the-art ultrasound machine by a highly experienced operator.

We have decided to publish this experience in spite of its limited size, as we think the outcomes achieved with our multimodal therapy with combined antioxidants in these patients may prove useful in urology and andrology practice, and we would welcome randomized controlled studies being carried out in the future to investigate the effectiveness of this treatment in a larger number of PD patients.

## Data Availability

The data presented in this study are available in this article.

## References

[1] Weidner W, Schroeder-Printzen I, Weiske WH, *et al*. Sexual dysfunction in Peyronie’s disease: an analysis of 222 patients without previous local plaque therapy. J Urol. 1997;157(1):325–8. 10.1016/s0022-5347(01)65370-9 8976290

[2] Pryor JP, Ralph DJ. Clinical presentations of Peyronie’s disease. Int J Impot Res. 2002;14(5):414–7. 10.1038/sj.ijir.3900877 12454695

[3] Nelson CJ, Diblasio C, Kendirci M, *et al*. The chronology of depression and distress in men with Peyronie’s disease. J Sex Med. 2008;5(8):1985–90. 10.1111/j.1743-6109.2008.00895.x 18554257

[4] Devine CJJ, Somers KD, Jordan GH. Proposal: trauma as a cause of Peyronie’s lesion. J Urol. 1997;157:285–90. 10.1016/s0022-5347(01)65361-8 8976281

[5] Somers KD, Dawson DM. Fibrin deposition in Peyronie’s disease plaque. J Urol. 1997;157(1):311–5. 8976287

[6] El-Sakka AI, Salabas E, Dinçer M. The pathophysiology of Peyronie’s disease. Arab J Urol. 2013;11(3):272–7. 10.1016/j.aju.2013.06.006 PMC4442979 26558092

[7] Sikka SC, Hellstrom WJG. Role of oxidative stress and antioxidants in Peyronie’s disease. Int J Impot Res. 2002;14(5):353–60. 10.1038/sj.ijir.3900880 12454686

[8] Paulis G, Romano G, Paulis L, *et al*. Recent pathophysiological aspects of Peyronie’s disease: role of free radicals, rationale, and therapeutic implications for antioxidant treatment—literature review. Adv Urol. 2017;2017:4653512. 10.1155/2017/4653512 PMC5514334 28744308

[9] Yousif A, Natale C, Hellstrom WJG. Conservative therapy for Peyronie’s disease: a contemporary review of the literature. Curr Urol Rep. 2021;22(2):6. 10.1007/s11934-020-01024-8 33420664

[10] Natale C, McLellan DM, Yousif A, *et al*. Review of intralesional collagenase clostridium histolyticum injection therapy and related combination therapies in the treatment of Peyronie’s disease (an update). Sex Med Rev. 2021;9(2):340–9. 10.1016/j.sxmr.2020.01.005 32199788

[11] Paulis G. Combination therapy (in the treatment of Peyronie’s disease). In: Cavallini G, Paulis G, editors. Peyronie’s disease. A comprehensive guide. Switzerland: Springer International Publishing; 2015. p. 97–105.

[12] Eri LM, Thomassen H, Brennhovd B, *et al*. Accuracy and repeatability of prostate volume measurements by transrectal ultrasound. Prostate Cancer Prostatic Dis. 2002;5(4):273–8. 10.1038/sj.pcan.4500568 12627211

[13] Lee JS, Chung BH. Transrectal ultrasound versus magnetic resonance imaging in the estimation of prostate volume as compared with radical prostatectomy specimens. Urol Int. 2007;78(4):323–7. 10.1159/000100836 17495490

[14] Gallo L, Sarnacchiaro P. Ten-year experience with multimodal treatment for acute phase Peyronie’s disease: a real life clinical report. Actas Urol Esp (Engl Ed). 2019;43:182–9. 10.1016/j.acuro.2018.08.005 30704797

[15] Paulis G, Barletta D, Turchi P, *et al*. Efficacy and safety evaluation of pentoxifylline associated with other antioxidants in medical treatment of Peyronie’s disease: a case–control study. Res Rep Urol. 2016;8:1. 10.2147/RRU.S97194. PMC4706125 26770906

[16] Paulis G, Cavallini G, De Giorgio G, *et al*. Long-term multimodal therapy (verapamil associated with propolis, blueberry, vitamin E and local diclofenac) on patients with Peyronie’s disease (chronic inflammation of the tunica albuginea). Results of a controlled study. Inflamm Allergy Drug Targets. 2013;12(6):403–9. 10.2174/1871528112666131205112432 24304332

[17] Brant WO, Dean RC, Lue TF. Treatment of Peyronie’s disease with oral pentoxifylline. Nat Clin Pract Urol. 2006;3(2):111–5. 10.1038/ncpuro0409 16470210

[18] Smith JF, Shindel AW, Huang YC, *et al*. Pentoxifylline treatment and penile calcifications in men with Peyronie’s disease. Asian J Androl. 2011;13(2):322–5. 10.1038/aja.2010.117 PMC3565600 21102473

[19] Payton S. Therapeutic benefit of pentoxifylline for Peyronie’s disease. Nat Rev Urol. 2010;7(5):237. 10.1038/nrurol.2010.53 20458801

[20] Alizadeh M, Karimi F, Fallah MR. Evaluation of verapamil efficacy in Peyronie’s disease comparing with pentoxifylline. Global J Health Sci. 2014;6(7):23–30. 10.5539/gjhs.v6n7p23 PMC4796342 25363175

[21] Ciociola F, Colpi GM. Peyronie’s disease: a “triple oxygenant therapy.” Arch Ital Urol Androl. 2013;85(1):36–40. 10.4081/aiua.2013.1.36 23695404

[22] Favilla V, Russo GI, Privitera S, *et al*. Combination of intralesional verapamil and oral antioxidants for Peyronie’s disease: a prospective, randomised controlled study. Andrologia. 2014;46(8):936–42. 10.1111/and.12178 24124921

[23] Paulis G, De Giorgio G. Complete plaque regression in patients with Peyronie’s disease. After multimodal treatment with antioxidants a report of 2 cases. Am J Case Rep. 2022;23:e936146. 10.12659/AJCR.936146. PMC9022185 35430575

[24] Kwon KD, Choi MJ, Park JM, *et al*. Silencing histone deacetylase 2 using small hairpin RNA induces regression of fibrotic plaque in a rat model of Peyronie’s disease. BJU Int. 2014;114(6):926–36. 10.1111/bju.12812 24841412

[25] Tal R, Hall MS, Alex B, Choi J, Mulhall JP. Peyronie’s disease in teenagers. J Sex Med. 2012;9(1):302–8. 10.1111/j.1743-6109.2011.02502.x 21981606

[26] Búfalo MC, Ferreira I, Costa G, *et al*. Propolis and its constituent caffeic acid suppress LPS-stimulated pro-inflammatory response by blocking NF-κB and MAPK activation in macrophages. J Ethnopharmacol. 2013;149(1):84–92. 10.1016/j.jep.2013.06.004 23770030

[27] Ebadi M, Sharma SK, Wanpen S, Amornpan A. Coenzyme Q10 inhibits mitochondrial complex-1 down-regulation and nuclear factor-kappa B activation. J Cell Mol Med. 2004;8:213–22. 10.1111/j.1582-4934.2004.tb00276.x PMC6740220 15256069

[28] Karlsen A, Paur I, Bøhn SK, *et al*. Bilberry juice modulates plasma concentration of NF-kappaB related inflammatory markers in subjects at increased risk of CVD. Eur J Nutr. 2010;49(6):345–55. 10.1007/s00394-010-0092-0 20119859

[29] Zhang DM, Guo ZX, Zhao YL, *et al*. l-Carnitine regulated Nrf2/Keap1 activation in vitro and in vivo and protected oxidized fish oil-induced inflammation response by inhibiting the NF-κB signaling pathway in Rhynchocypris lagowski Dybowski. Fish Shellfish Immunol. 2019;93:1100–10. 10.1016/j.fsi.2019.08.041 31422179

[30] Gülçin I. Antioxidant and antiradical activities of l-carnitine. Life Sci. 2006;78(8):803–11. 10.1016/j.lfs.2005.05.103 16253281

[31] Mohamed HA, Said RS. Coenzyme Q10 attenuates inflammation and fibrosis implicated in radiation enteropathy through suppression of NF-kB/TGF-β/MMP-9 pathways. Int Immunopharmacol. 2021;92: 107347. 10.1016/j.intimp.2020.107347. 33418245

[32] Tian MY, Fan JH, Zhuang ZW, *et al*. Effects of silymarin on p65 NF-κB, p38 MAPK and CYP450 in LPS-induced hoof dermal inflammatory cells of dairy cows. BMC Vet Res. 2019;15(1):127. 10.1186/s12917-019-1868-y PMC6492397 31039823

[33] Wang Y, Wang R, Wang Y, *et al*. Ginkgo biloba extract mitigates liver fibrosis and apoptosis by regulating p38 MAPK, NF-κB/IκBα, and Bcl-2/Bax signaling. Drug Des Dev Ther. 2015;9:6303–17. 10.2147/DDDT.S93732 PMC4671772 26664050

[34] Wang WG, Xiu RJ, Xu ZW, *et al*. Protective effects of Vitamin C against spinal cord injury-induced renal damage through suppression of NF-κB and proinflammatory cytokines. Neurol Sci. 2015;36(4):521–6. 10.1007/s10072-014-1965-4 25403314

[35] Godbout JP, Berg BM, Krzyszton C, Johnson RW. Alpha-tocopherol attenuates NFkappaB activation and pro-inflammatory cytokine production in brain and improves recovery from lipopolysaccharide-induced sickness behavior. J Neuroimmunol. 2005;169(1–2):97–105. 10.1016/j.jneuroim.2005.08.003 16146653

[36] Costantini TW, Deree J, Peterson CY, *et al*. Pentoxifylline modulates p47phox activation and downregulates neutrophil oxidative burst through PKA-dependent and -independent mechanisms. Immunopharmacol Immunotoxicol. 2010;32(1):82–91. 10.3109/08923970903183557 19839729

[37] Sunil VR, Vayas KN, Cervelli JA, *et al*. Pentoxifylline attenuates nitrogen mustard-induced acute lung injury, oxidative stress and inflammation. Exp Mol Pathol. 2014;97(1):89–98. 10.1016/j.yexmp.2014.05.009 PMC4271840 24886962

[38] Freitas JP, Filipe PM. Pentoxifylline. A hydroxyl radical scavenger. Biol Trace Elem Res. 1995;47(1–3):307–11. 10.1007/BF02790131 7779563

[39] Ji Q, Zhang L, Jia H, Xu J. Pentoxifylline inhibits endotoxin-induced NF-kappa B activation and associated production of proinflammatory cytokines. Ann Clin Lab Sci. 2004;34(4):427–36. 15648785

[40] Jin F, Li X, Lee HJ, Lee CJ. diclofenac inhibits phorbol ester-induced gene expression and production of MUC5AC mucin via affecting degradation of IkBα and translocation of NF-kB p65 in NCI-H292 cells. Biomol Ther (Seoul). 2020;28(5):431–6. 10.4062/biomolther.2020.090 PMC7457166 32753566

[41] Radermacher J, Jentsch D, Scholl MA, *et al*. Diclofenac concentrations in synovial fluid and plasma after cutaneous application in inflammatory and degenerative joint disease. Br J Clin Pharmacol. 1991;31(5):537–41. 10.1111/j.1365-2125.1991.tb05576.x PMC1368472 1888621

[42] Hatzimouratidis K, Eardley I, Giuliano F, *et al*. EAU guidelines on penile curvature. Eur Urol. 2012;62(3):543–52. 10.1016/j.eururo.2012.05.040 22658761

[43] McCauley JF, Dean RC. Diagnostic utility of penile ultrasound in Peyronie’s disease. World J Urol. 2020;38(2):263–8. 10.1007/s00345-019-02928-y 31606787

[44] Parmar M, Masterson JM, Masterson TA 3rd. The role of imaging in the diagnosis and management of Peyronie’s disease. Curr Opin Urol. 2020;30(3):283–9. 10.1097/MOU.0000000000000754 32205808

